# The Complications of Scarlet Fever

**Published:** 1910-08-20

**Authors:** 


### Medicine.

### THE COMPLICATIONS OF SCARLET FEVER.

Every specific fever seems to exhibit periodic variations in its virulence, the length of each interval between one time of maximum intensity and the next being measured in years. Thirty years ago scarlet fever was very virulent; now it is comparatively mild; in another twenty or thirty years, or possibly sooner, it will very likely be formidable again. This long-drawn-out periodic variation in the intensity of any disease necessitates revision of the statements made about it from time to time. This is not peculiar to scarlet fever; it is probably true of all specific or microbic maladies. It is certainly true of lobar pneumonia, for instance. In some pneumococcal epidemics empyema is common, in others it is rare; in some years the mortality from pneumonia is high, in others low. What is true about the effects of a disease at one time is not necessarily true at all times therefore, and it is of interest to note what, in their order of percentage incidence, the complications of scarlet fever are to-day. These can be gathered from the published statistics of the Metropolitan Asylums Board for the year 1909. Altogether 16,191 cases of scarlatina were dealt with, and the following list of the complications speaks for itself:—

| | Number of cases out of the total 16,191. | Percentage incidence. |
|---|---|---|
| Otitis Media | 2,056 | 12.70 |
| Albuminuria, including all cases in which albumin was detected, and in which there were no other signs of nephritis, even if only on one occasion | 1,510 | 9.32 |
| Adenitis of convalescence, simple | 1,245 | 7.68 |
| Nephritis, definite; distinct from albuminuria above | 909 | 5.61 |
| Rheumatism | 628 | 3.88 |
| Relapse of the disease | 322 | 1.98 |
| Tonsilitis during convalescence | 263 | 1.65 |
| Adenitis of convalescence, suppurative | 205 | 1.26 |
| Abscesses, exclusive of mastoid or cervical abscesses | 173 | 1.07 |
| Stomatitis, ulcerative | 156 | 0.96 |
| Adenitis, suppurative, occurring in the acute stage | 135 | 0.83 |
| Endocarditis | 118 | 0.73 |
| Mastoid abscess, including all cases with abscess formation in or around the mastoid bone | 105 | 0.65 |
| Broncho-pneumonia | 85 | 0.52 |
| Bronchitis | 76 | 0.47 |
| Jaundice | 50 | 0.31 |
| Pneumonia | 42 | 0.26 |
| Cervical cellulitis | 41 | 0.25 |
| Laryngitis | 22 | 0.13 |
| Pleurisy | 18 | 0.11 |
| Empyema | 17 | 0.10 |
| Pyæmia | 15 | 0.09 |
| Chorea | 13 | 0.08 |
| Pericarditis | 11 | 0.07 |
| Meningitis | 10 | 0.06 |

It will be seen that otitis media is by far the commonest complication, and that albuminuria, adenitis, nephritis, and rheumatism are also prominent risks.

### Other Fevers Complicating Scarlatina.

It is a remarkable thing how one specific fever seems to predispose to another. Even though one can find no trace of how the infection has reached the patient, it is surprising how chicken-pox is apt to come directly after measles, mumps after measles or whooping-cough, and so on. It is as true as almost any other aphorism to say that two things should never be omitted from the list of complications or sequelæ of any specific fever: (1) General tuberculosis, and (2) another specific fever.

The importance of this lies in the fact that the second fever often has an unwonted severity when it occurs in a child that is already debilitated by another febrile illness from which convalescence is not yet complete. Chicken-pox, for instance, is seldom serious when it develops in a child that has hitherto been strong and well; but if it occurs during convalescence from, say, measles it may be very serious indeed. Similarly, if diphtheria develops before complete recovery from scarlet fever the outlook is grave; and so on. It is probable that isolation in special hospitals, from which are excluded visitors who may be carrying the infection of mumps, measles, etc., has much to do with the comparative scarcity with which patients admitted with scarlet fever under the care of the Metropolitan Asylums Board suffer from another specific fever at the same time or soon afterwards, and this must be an additional factor in diminishing the mortality of the disease. It would be interesting to compare the percentage incidence of co-existent infectious diseases in scarlatina cases treated (*a*) at home and (*b*) in special isolation hospitals respectively. Unfortunately it is almost impossible to get records of any large number of cases treated at home, and one has to be content with the published statistics of the Metropolitan Asylums Board, which for the year 1909 are as follows:—

| Infectious disease co-existent with scarlet fever. | Number of cases out of a total of 16,191. | Percentage incidence. |
|---|---|---|
| Diphtheria | 299 | 1.85 |
| Chicken-pox | 214 | 1.32 |
| Measles | 211 | 1.30 |
| Whooping-cough | 101 | 0.62 |
| Mumps | 29 | 0.18 |
| German Measles | 26 | 0.16 |
| Erysipelas | 11 | 0.07 |
| Typhoid Fever | 2 | 0.01 |

It is extremely probable that in almost all these cases the double infection had occurred before the patient was transferred to the isolation hospital. Indeed, it is not at all uncommon, not merely for a person admitted with scarlatina to develop another specific fever while in the hospital, but even for two, or possibly three, different infectious diseases to be actually present simultaneously in the same case on admission. The figures given herewith indicate how frequently the following diseases were co-existent on admission in the case of the Metropolitan Asylums Board in 1909:—

| | |
|---|---|
| Scarlet fever and Chicken-pox | in 146 cases |
| Scarlet fever and Diphtheria | 109 „ |
| Scarlet fever and Whooping-cough | 104 „ |
| Diphtheria and Measles | 100 „ |
| Scarlet fever and Measles | 58 „ |
| Diphtheria and Whooping-cough | 46 „ |
| Diphtheria and Chicken-pox | 21 „ |

